# Morphological and molecular identification of two new *Alternaria* species (Ascomycota, Pleosporaceae) in section Radicina from China

**DOI:** 10.3897/mycokeys.78.64853

**Published:** 2021-04-09

**Authors:** Lin He, Hong Cheng, Lin Zhao, Aye Aye Htun, Zhi He Yu, Jian Xin Deng, Qi Li Li

**Affiliations:** 1 Department of Plant Protection, College of Agriculture, Yangtze University, Jingzhou 434025, China Yangtze University Jingzhou China; 2 Forewarning and Management of Agricultural and Forestry Pests, Hubei Engineering Technology Center, Yangtze University, Jingzhou 434025, China Institute of Plant Protection, Guangxi Academy of Agricultural Sciences and Guangxi Key Laboratory of Biology for Crop Diseases and Insect Pests Nanning China; 3 Department of Applied Microbiology, College of Life Sciences, Yangtze University, Jingzhou 434025, China Yangtze University Jingzhou China; 4 Institute of Plant Protection, Guangxi Academy of Agricultural Sciences and Guangxi Key Laboratory of Biology for Crop Diseases and Insect Pests, Nanning, Guangxi, 530007, China Institute of Plant Protection, Guangxi Academy of Agricultural Sciences and Guangxi Key Laboratory of Biology for Crop Diseases and Insect Pests Nanning China

**Keywords:** *
Alternaria
*, new taxon, phylogeny, Pleosporaceae, taxonomy

## Abstract

The fungal genus *Alternaria* was distributed widely and found in different habitats such as plant or indoor environment. During an investigation into this genus in China, two new *Alternaria* species, *Alternaria vulgarae* and *A. divaricatae* were respectively isolated from diseased leaves of *Foeniculum vulgare* and *Saposhnikovia divaricata*, which both belonged to Umbelliferae. Phylogenetically, they were determined as new species belonging in the section Radicina of *Alternaria* based on the combined four gene fragments of ITS, *TEF1*, *GAPDH* and *RPB2*. Morphologically, the two species were illustrated and compared with other relevant Alternaria species in section Radicina.

## Introduction

*Alternaria* Nees (1816) was typified by *Alternaria tenuis* (the synonym of *A. alternata*), a species with muriform and catenulate conidia. Since then, hundreds of new species were proposed in the genus. Meanwhile, because of unstable taxonomic standards (morphological characteristics, host and growing environment, etc.), the controversies about species boundary started and never stopped (Elliott 1917; Fries 1832; Neergaard 1945; Joly 1964; Wiltshire 1933; Simmons 1967, 1992). In 1992, Simmons introduced reasonable standards to get unified taxonomic concepts on *Alternaria* species based on colony and conidial morphology. At the same time, the concept of species-group was introduced, the small-spored, catenulate taxa of *Alternaria* were divided into six morphological groups by Simmons and Roberts (1993). More recently, around 300 *Alternaria* morphospecies have been accepted based on the shape, size, septation of conidia, as well as sporulation patterns. Small-spored *Alternaria* species were also redefined and divided into 10 subsections characterized by short (>50(–60) μm) or medium (50–100(–105) μm) conidia produced in various patterns of branched and unbranched chains or solitary (Simmons 2007). However, the identification remained challenging due to the impact of environmental conditions and other unknown factors.

On the other hand, multigene phylogenetic analyses have provided strong support for the re-definition of the *Alternaria* genus. Many sequences of gene regions such as the internal transcribed spacer region of rDNA (ITS), large subunit ribosomal DNA (*LSU*), mitochondrial small subunit (mtSSU), *Alternaria* major allergen (*ALT*), glyceraldehydes-3-phosphate dehydrogenase (*GAPDH*), translation elongation factor 1-alpha (*TEF1*), RNA polymerase second largest subunit (*RPB2*), and ATPase etc. were applied to delimit the genus (Pryor and Gilbertson 2000; Hong et al. 2005; Lawrence et al. 2012, 2013; Woudenberg et al. 2013, 2014; Poursafar et al. 2018). In recent studies, both morphological and molecular analyses were used for the delimitation of the genus *Alternaria*, which has been divided into 28 sections and eight monotypic lineages (Woudenberg et al. 2013; Lawrence et al. 2016; Ghafri et al. 2019; Marin-Felix et al. 2019). The number of *Alternaria* species has been continuously growing after re-descriptions and new discovery (Deng et al. 2018; Ahmadpour 2019: Liu et al. 2019; Tao et al. 2019; Bessadat et al. 2020; He et al. 2020). Coincidentally, several phylogenetic lineages have strongly supported morphology-based sections but others not (Simmons 2007; Woudenberg et al. 2015).

During the investigation into *Alternaria* species in China, two new taxa were isolated from umbelliferous plants, *Foeniculum vulgare* and *Saposhnikovia divaricata*. The study was designed to determine them based on a polyphasic approach including morphology and phylogenetic analyses.

## Materials and methods

### Isolation and morphological studies

Leaves of *Foeniculum vulgare* and *Saposhnikovia divaricata* with necrotic spots were respectively collected from Wenjiang district (Chengdu, Sichuan in June, 2015) and Badong county (Yichang, Hubei in July, 2016) in China. For fungal isolation, the samples were stored in sterile plastic bags and transported to the laboratory. The tissues were cut into small segments and placed on moist filter papers within Petri dishes then incubated at 25 °C to stimulate sporulation. After 24 h, the samples were examined under a stereomicroscope. *Alternaria*-like spores were picked up and inoculated to potato dextrose agar (PDA: Difco, Montreal, Canada) using sterilized glass needles. All isolated pure cultures were inoculated to test-tube slants and stored at 4 °C. Dried cultures from the single spore and ex-type strains were deposited in the Fungi Herbarium of Yangtze University (YZU), Jingzhou, Hubei, China.

To determine colonial characteristics (size, color and texture of colony), the strains were cultured on PDA at 25 °C for 7 days in darkness. To analyze the morphological features of conidia (conidial size, shape, sporulation, etc.), fresh mycelia were transferred on potato carrot agar (PCA) and V8 juice agar (V8A) then incubated at 22 °C under an 8 hour photoperiod for 7 days (Simmons 2007). Conidia were mounted into a lactophenol picric acid solution and digital images were captured under a Nikon ECLIPSE Ni-U microscope system (Nikon, Japan). Conidia (n = 50) were randomly selected for determining the morphology and sporulation patterns were also photographed at the same time.

### DNA extraction, PCR amplification and sequencing

Genomic DNA was extracted from fresh mycelia growing on PDA after 3–5 days of growth following the CTAB method described in Watanabe et al. (2010). For amplification of the ITS, *TEF1*, *GAPDH* and *RPB2* gene fragments, the primer pairs ITS5/ITS4 (White et al. 1990), EF1-728F/EF1-986R (Carbone and Kohn 1999), gpd1/gpd2 (Berbee et al. 1999) and RPB2-5F/RPB2-7cR (Liu et al. 1999) were used, respectively. A total of 25 μL of a PCR reaction mixture containing 21μL of 1.1×Taq PCR Star Mix (TSINGKE, Beijing, China), 2 μL template DNA and 1μL of each primer was prepared and the PCR was performed in an Eppendorf Mastercycler following the protocols described by Woudenberg et al. (2013). Successful products were purified and sequenced by TSINGKE company (Beijing, China). All sequences were assembled with BioEdit (Hall 1999) and deposited at GenBank (https://www.ncbi.nlm.nih.gov/) (Table 1).

**Table 1.** *Alternaria* strains and their accession numbers used in the phylogenetic analysis.

**Section**	**Species**	**Strain**	**Host/Substrate**	**Country**	**GenBank accession numbers**			
					**ITS**	***GAPDH***	***TEF1***	***RPB2***
* Alternaria *	*A. alternata*	CBS 916.96 T	*Arachis hypogaea*	India	AF347031	AY278808	KC584634	KC584375
	*A. tenuissima*	CBS 918.96 R	*Dianthus* sp.	UK	AF347032	AY278809	KC584693	KC584435
* Althernantherae *	*A. alternantherae*	CBS 124392	*Solanum melongena*	China	KC584179	KC584096	KC584633	KC584374
	*A. perpunctulata*	CBS 115267 T	*Alternanthera philoxeroides*	USA	KC584210	KC584129	KC584676	KC584418
* Gypsophilae *	*A. gypsophilae*	CBS 107.41 T	*Gypsophila elegans*	USA	KC584199	KC584118	KC584660	KC584401
	*A. nobilis*	CBS 116490 R	*Dianthus caryophyllus*	New Zealand	KC584208	KC584127	KC584673	KC584415
	*A. vaccariae*	CBS 116533 R	*Vaccaria hispanica*	USA	KC584223	KC584146	KC584696	KC584438
	*A. vaccariicola*	CBS 118714 T	*Vaccaria hispanica*	USA	KC584224	KC584147	KC584697	KC584439
* Radicina *	*A. carotiincultae*	CBS 109381 T	*Daucus carota*	USA	KC584188	KC584106	KC584645	KC584386
	*A. chlamydosporifera*	FMR 17360 T	Rabbit dung	Spain	LR133924	LR133927	LR133929	LR133926
	***A. divaricatae* sp. nov.**	**YZU 151055 T**	***Saposhnikovia divaricata***	**China**	**MW541932**	–	**MW579314**	**MW579316**
		**YZU 151059**	***Saposhnikovia divaricata***	**China**	**MW541933**	–	**MW579315**	**MW579317**
	*A. glehniae*	YZU 161149 T	*Glehnia littoralis*	China	MK279385	–	MK279392	MK279394
	*A. petroselini*	CBS 112.41 T	*Petroselinum sativum*	Unknown	KC584211	KC584130	KC584677	KC584419
	*A. radicina*	CBS 245.67 T	*Daucus carota*	USA	KC584213	KC584133	KC584681	KC584423
	*A. selini*	CBS 109382 T	*Petroselinum crispum*	Saudi Arabia	AF229455	AY278800	KC584684	KC584426
	*A. smyrnii*	CBS 109380 R	*Smyrnium olusatrum*	UK	AF229456	KC584138	KC584687	KC584429
	***A. vulgarae* sp. nov.**	**YZU 161234 T**	***Foeniculum vulgare***	**China**	**MW541936**	**MW579308**	**MW579310**	**MW579312**
		**YZU 161235**	***Foeniculum vulgare***	**China**	**MW541937**	**MW579309**	**MW579311**	**MW579313**
* Porri *	*A. dauci*	CBS 117097 R	*Daucus carota*	USA	KC584192	KC584111	KC584651	KC584392
	*A. porri*	CBS 116698 R	*Allium cepa*	USA	DQ323700	KC584132	KC584679	KC584421
* Sonchi *	*A. cinerariae*	CBS 116495 R	*Ligularia* sp.	USA	KC584190	KC584109	KC584648	KC584389
	*A. sonchi*	CBS 119675 R	*Sonchus asper*	Canada	KC584220	KC584142	KC584691	KC584433
Out-group	*Stemphylium herbarum*	CBS 191.86 T	*Medicago sativa*	India	KC584239	AF443884	KC584731	KC584471

Notes: *Alternaria* strains of the present study are marked in bold. Type strains are marked ‘T’. Representative strains are marked ‘R’.

### Phylogenetic analyses

Preliminary BLAST searches in GenBank with ITS and *TEF1* sequences of the present isolates indicated that they had a close phylogenetic relationship with species from section Radicina of *Alternaria*. Subsequently, sequence data of 19 *Alternaria* species and *Stemphylium herbarum* CBS 191.86 (outgroup) were retrieved from National Center for Biotechnology Information (NCBI), mostly published in Marin-Felix et al. (2019), Woudenberg et al. (2013), and Tao et al. (2019) (Table 1). The gene sequences were concatenated and edited manually according to ITS+*TEF1*+*GAPDH*+*RPB2* for YZU 161234 and YZU 161235 and ITS+*TEF1*+*RPB2* for YZU 151055 and YZU 151059 with equal weight in MEGA v.7.0.26 (Kumar et al. 2016). Maximum parsimony (MP) analysis was performed in PAUP 4.0 (Swofford 2002) using the heuristic search option of 1000 random-addition sequences and tree bisection and reconnection (TBR) as the branch-swapping algorithm. Gaps were treated as missing data. The bootstrap values (BS) with 1000 replicates were performed to determine branch support. Parsimony scores of tree length (TL), consistency index (CI), retention index (RI) and rescaled consistency (RC) were calculated for each generated tree. The Bayesian inference (BI) analysis was performed with a Markov Chain Monte Carlo (MCMC) algorithm with Bayesian posterior probabilities in MrBayes v. 3.2.1 (Ronquist et al. 2012). The best-fit evolutionary models (GTR+I+G) were determined in MrModel-test v. 2.3 (Posada and Crandall 1998) using the Akaike Information Criterion (AIC). Two independent analyses with four chains each were run for 10,000,000 generations. Trees were sampled every 100^th^ generation. The run was stopped until the standard deviation of split frequencies reaches < 0.01 and the initial 25 % of the trees were discarded as the burn-in phase of each analysis. Maximum likelihood (ML) analysis was performed using RAxML v.7.2.8 (Stamatakis 2006), implementing GTRCAT model and executing 1000 rapid ML bootstrap replications. Branch support equal to or above 0.70/70%/70% for PP (posterior probability of BI analysis) and BS (bootstrap for ML and MP analyses) values were shown at the nodes in the phylogram.

## Results

### Phylogenetic analyses

**Figure 1.**
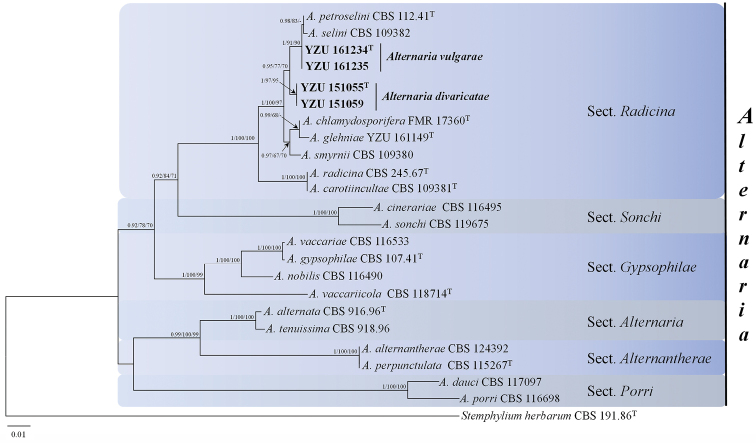
Phylogenetic tree based on the combined gene sequences of ITS, *TEF1*, *GAPDH*, and *RPB2*. The Bayesian posterior probabilities >0.70 (PP), maximum likelihood and maximum parsimony bootstrap support values >70 (BS) are given at the nodes (PP/BS). Examined strains are in bold.

The combined dataset of twenty-four strains (including 20 references and present four strains) had a length of 2166 characters with gaps after alignment, 536 characters for ITS, 247 for *TEF1*, 537 for *GAPDH* and 846 for *RPB2*. Of these characters, 1555 were constant and 198 were variable and parsimony-uninformative. MP analysis of the remaining 413 parsimony-informative characters resulted in one parsimonious tree of 995 lengths (CI = 0.739, RI = 0.815, RC = 0.602); Tree topologies computed from the MP, BI, and ML analysis were similar and the ML tree was shown in Fig. 1. The results indicated that all strains in the present study fell into the section Radicina with PP/BS (BI/ML/MP) values of 1/100%/100%. The strains YZU 161234 and YZU 161235 were clustered with *A. petroselini* and *A. selini* in a clade supported by values of 1.0/91%/90% (BI/ML/MP). This clade was sister to a separate clade containing the other two strains (YZU 151055, YZU 151059) supported by PP value of 0.95 and BS values of 77%/70% (ML/MP) (Fig. 1).

### Taxonomy

#### Alternaria divaricatae

Taxon classificationFungiPleosporalesPleosporaceae

L. He & J.X. Deng sp. nov.

42E5E682-C708-57EE-8001-7DDD3A2EFD75

838893

Figure 2

##### Type.

China, Sichuan Province, Chengdu City, Wenjiang District, Herb Garden of Chengdu University of Traditional Chinese Medicine, from leaf spot of *Saposhnikovia divaricata*. 17 June, 2015, J.X Deng, (YZU-H-0029, holotype), ex-type culture YZU 151055.

##### Etymology.

In reference to the host species name, *divaricata*.

##### Description.

**Figure 2.**
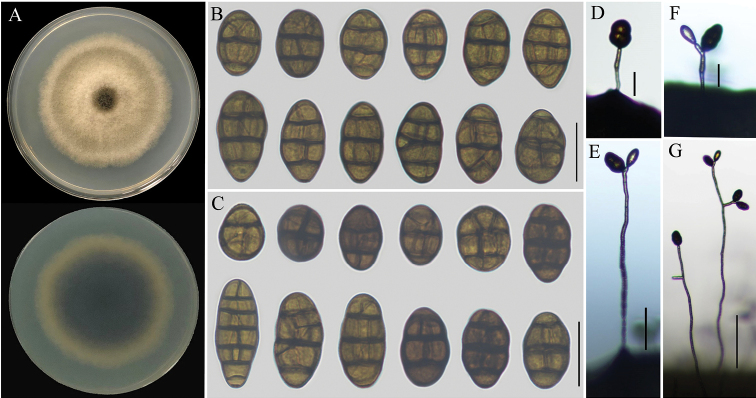
Morphological characteristics of *Alternaria divaricatae* (strain: YZU 151055). Colony on PDA for 7 days at 25 °C (**A**); Conidia on PCA and V8A (**B, C**); Sporulation patterns from PCA and V8A (**D–G**: **D, E** from PCA**F, G** from V8A); Scale bars: 25 µm (**B, C**); 2 µm (**D, F**); 50 µm (**E**); 100 µm (**G**).

*Colonies* on PDA (Fig. 2A) vinaceous buff, hazel in the center, velvety, cottony, dark mouse grey to pale mouse grey in reverse, 56‒64 mm in diam.; On PCA, *conidiophores* arising directly from lateral or apical of aerial hyphae or medium, lightly flexuous, sometimes geniculate at apex, smooth-walled, 9–36 × 3.5–6 μm, 1–3 transverse septa, the aerial hyphae sometimes up to 82–400 × 4–6 μm; *conidia* solitary from apex or geniculate loci, short-ovoid, subglobose, ellipsoid, 21–38 × 12–26 μm, with 1–4 transverse septa and 1‒4 longitudinal septa (Fig. 2B, D, E); On V8A, *conidiophores* 10–26 (–53) × 3–4 μm, 1‒7 transverse septa, *conidia* 22–39 × 13–24 μm, 1‒4 transverse septa, 1‒3 longitudinal or oblique between septa (Fig. 2C, F, G). There was no secondary conidium production observed on PCA and V8A medium.

##### Additional isolate examined.

China, Sichuan Province, Chengdu City, Wenjiang District, Herb Garden of Chengdu University of Traditional Chinese Medicine, from leaf spot of *Saposhnikovia divaricata*. 17 June, 2015, L He, living culture YZU 151059.

##### Notes.

Phylogenetically, *Alternaria divaricatae* forms a distinct clade in section Radicina, which appears to be sister to a clade including *A. petroselini*, *A. selini* and *A. vulgarae* (Fig. 1). Morphologically, *A. divaricatae* was different from *A. petroselini*, *A. selini* and *A. vulgarae* by producing smaller conidia (Table 2) and special sporulation from apex or geniculate loci of lateral or apical of aerial hyphae. Moreover, *A. chlamydosporifera*, *A. glehniae* and *A. smyrnii* grouped together and clustered as a sister clade with *A. divaricatae*, *A. petroselini*, *A. selini* and *A. vulgarae* (Fig. 1). Obviously, the conidia of *A. divaricatae* was smaller than *A. smyrnii* (Table 2) and *A. divaricatae* could be also easily differentiated from *A. chlamydosporifera* by the lack of chlamydospores in culture (Marin-Felix et al. 2019). Meanwhile, *A. glehniae* was distinguished from *A. divaricatae* by its single conidium on apex of conidiophore (there was no geniculate sporulation loci) and production of secondary conidium (Tao et al. 2019). In addition, *A. radicina* and *A. carotiincultae* were distinguished from present species by distant phylogenetic relationship in section Radicina.

**Table 2.** Morphological comparison of the present species and other Altenraria species in section Radicina

Species	Conidia			Conidia per chain	Medium
	Shape	Size (μm)	Septa		
*A. atrocariis*	Ovoid, ellipsoid	50–100×25–38	3–12	1–2	Host^a^
***A. divaricatae* sp. nov.**	**Short-ovoid,subglobose, ellipsoid**	**21–38×12–26**	**1–4**	**1**	**PCA^d^**
		**22–39×13–24**	**1–4**		**V8A^d^**
*A. carotiincultae*	Long ovoid or ellipsoid	40–80×15–23	5–7 (–11)	1–3	PCA ^a^
*A. chlamydosporifera*	ellipsoidal or ovoid, occasionally, subglobose	12–41×7–20	1–3(–4)	1, occasionally 2	PCA ^b^
*A. glehniae*	Long ovoid, ellipsoid	20‒40 (–48)×10‒20	3–7	1, occasionally 2	PCA ^c^
*A. petroselini*	Short-ovoid to subsphaeroid	35–62(‒66)×20–26	6–8	1, rarely to 2	PCA ^a^
*A. radicina*	Short-broad or long-narrow ellipsoid and ovoid	42–63×15–20	4–8	1, seldom up to 2	PCA ^a^
*A. selini*	Short-ovoid	32–42(–50)×22–27	3–5	1–3	PCA ^a^
	Long-ellipsoid	48–65(–50)×15–20	Up to 7		
*A. smyrnii*	Ovoid, obovoid	40–58×18–22	7–8(–10)	1–2	PCA ^a^
	Narrower ellipsoid	67–96×13–16			
***A. vulgarae* sp. nov.**	**Short-ovoid, ovoid or long-ellipsoid**	**25–50 (–70)×16–27**	**1–5**	**1**	**PCA^d^**
		**24–55 (–77)×13–26**	**1–8**		**V8A^d^**

^a^ referenced from Simmons (2007); ^b^ referenced from Marin-Felix et al. (2019); ^c^ referenced from Tao et al. (2019); ^d^ determined in the present study.

#### Alternaria vulgarae

Taxon classificationFungiPleosporalesPleosporaceae

L. He & J.X. Deng sp. nov.

FDC16883-D5D6-5064-AC0C-A9C487854856

838892

Figure 3

##### Type.

China, Hubei Province, Yichang city, Badong county on infected leaves of *Foeniculum vulgare*. 19 July, 2016, J.X Deng, (YZU-H-0040, holotype), ex-type culture YZU 161234.

##### Etymology.

In reference to the host species name, *vulgare*.

##### Description.

**Figure 3.**
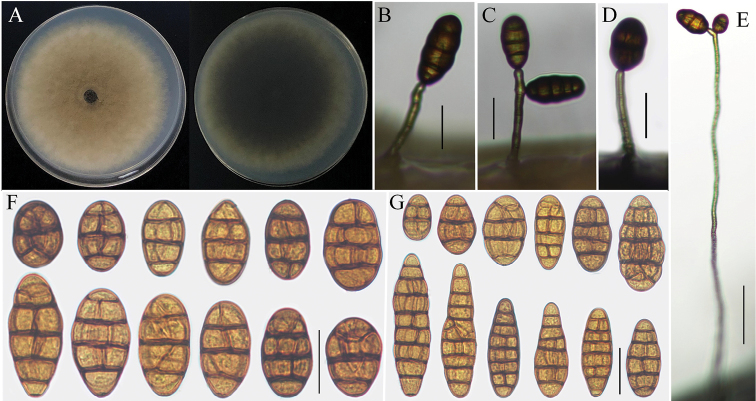
Morphological characteristics of *Alternaria vulgarae* (strain: YZU 161234). Colony on PDA for 7 days at 25 °C (**A**); Sporulation patterns on PCA and V8A (**B–E**: **B, C** from V8A**D, E** from PCA); Conidia from PCA and V8A (**F–G**). Scale bars: 25 µm (**B, C, D, F, G**); 50 µm (**E**).

*Colonies* on PDA (Fig. 3A) hazel in center and vinaceous buff at the edge, greenish black to mouse gray in reverse, surface velvety or floccose, 79‒82 mm in diam.; On PCA, *conidiophores* straight or curved, 12–80 × 4–6 μm, 1‒4 transverse septa; *conidia* solitary arising from the apex or near the apex of the conidiophores or terminal hyphae, rare from lateral of wire-like hyphae, ovoid, short-ovoid or ellipsoid, 25–50 (–70) × 16–27 μm, with 1–5 transverse septa and 1‒4 longitudinal septa (Fig. 3B, C, F); On V8A, *conidiophores* 24–93 × 4–7 μm, 1‒4 transverse septa, wire-like hyphae up to 200–400 × 4–6 μm; *conidia* short-ovoid, ovoid, ellipsoid or long-ellipsoid, 24–55 (–77) × 13–26 μm, 1‒8 transverse septa, 1‒4 longitudinal or oblique between septa (Fig. 3D, E, G). There was no secondary conidium production observed on PCA and V8A medium.

##### Additional isolate examined.

China, Hubei Province, Yichang city, Badong county on infected leaves of *Foeniculum vulgare*. 19 July, 2016, L He, living culture YZU 161235.

##### Notes.

Phylogenetic analysis based on combining four gene fragments indicated that *Alternaria vulgarae* fell in an individual branch in section Radicina of *Alternaria* and displayed a close relationship with *A. petroselini* and *A. selini* with high supported values (Fig. 1). Morphologically, *A. vulgarae* could be easily distinguished from *A. petroselini* and *A. selini* by their sporulation and length of conidiophores. Conidia of *A. petroselini* were solitary or cluster a small clump with 2‒4 spores near the tips or lateral of conidiophores. Occasionally, the secondary conidium could be observed. Meanwhile, the single conidium or conidial chains (1–3) of *A. selini* grew from numerous lateral conidiophores, which produced from wire-like hyphae (Simmons 2007). Differently, conidia of *A. vulgarae* were erected from apex of conidiophores or terminal hyphae. There were no small conidial clumps and secondary conidium formed (Fig. 3B, C, D, E). Moreover, the conidiophores of *A. vulgarae* (12–80 × 4–6 μm) was longer than *A. petroselini* (30–60 × 5–6.5 μm) and shorter than *A. selini* (200–400 × 4–6 μm) (Simmons 2007). Besides, *A. vulgarae* differed from *A. petroselini* in conidial shape. Conidium populations of *A. petroselini* were dominated by shot-ovoid to subsphaerical spores though, the shapes of *A. vulgarae* were mainly ovoid, ellipsoid or long-ellipsoid (Simmons 2007).

## Discussion

Morphologically, *Alternaria radicina* species-group was one of the 10 subsections (A–1) and comprised 8 species described by Simmons (2007): *A. atrocariis*, *A. carotiincultae*, *A. japonica*, *A. petroselini*, *A. radicina*, *A. selini*, *A. smyrnii* and *A. soliaridae*. With the development of molecular studies, the species-group was re-defined and the section Radicina was introduced and perfected (Pryor and Gilbertson 2000; Lawrence et. al 2013; Woudenberg et al. 2013). Uniformly, species in this section had some similar morphological characters, such as conidiophores, sporulation, conidial shape and etc. The phylogenetic analysis showed that only five species were clustered in section Radicina. Except for *A. atrocariis*, which had no published sequence data, the two other species were shown to belong to other sections: *A. japonica* felled in the section Japonicae and *A. soliaridae* formed a separate monophyletic lineage (Woudenberg et al. 2013). Recently, two more species *A. chlamydosporifera* and *A. glehniae* were reported in the section Radicina (Marin-Felix et al. 2019; Tao et al. 2019).

In the current study, two new Alternaria species belonged to the section Radicina based on morphological and phylogenetic analysis. *Alternaria divaricatae* was identified as a novel species based on unique morphological and well-supported phylogenetic analysis (Fig. 2 and Table 2). Phylogenetically, *A. vulgarae* clustered with *A. petroselini* and *A. selini*. Although its phylogenetic position was not well-supported, *A. vulgarae* can be distinguished from these two species in section Radicina by morphological characteristics (Table 2). Except the length of conidiophores, *A. vulgarae* was characterized by its sporulation. Meanwhile, *A. vulgarae* won’t form secondary conidium (Table 2). These characters were important standards to identify *Alternaria* species (Simmons 2007). And, according to Jeewon and Hyde (2016), a fungal species can be defined based on the distinctive morphological characters even though the phylogenies were not well-supported, because the phylogeny cannot really reflect all morphologies.

## Supplementary Material

XML Treatment for Alternaria divaricatae

XML Treatment for Alternaria vulgarae
